# Customizable Ceramic Nanocomposites Using Carbon Nanotubes

**DOI:** 10.3390/molecules24173176

**Published:** 2019-09-01

**Authors:** Chinyere Okolo, Rafaila Rafique, Sadia Sagar Iqbal, Tayyab Subhani, Mohd Shahneel Saharudin, Badekai Ramachandra Bhat, Fawad Inam

**Affiliations:** 1 Department of Mechanical and Construction Engineering, Northumbria University, Newcastle upon Tyne NE1 8ST, UK; 2 H.E.J. Research Institute of Chemistry, International Centre for Chemical and Biological, Sciences, University of Karachi, Karachi 75270, Pakistan; 3 Department of Physics, University of Lahore, Lahore 54590, Pakistan; 4 Department of Mechanical Engineering, University of Hail, Hail 81451, Saudi Arabia; 5 Malaysia Italy Design Institute (UniKL MIDI), Universiti Kuala Lumpur, Kuala Lumpur 59100, Malaysia; 6 Department of Chemistry, National Institute of Technology Karnataka, Mangaluru 575025, India; 7 Department of Engineering and Computing, University of East London, London E16 2RD, UK

**Keywords:** ceramic nanocomposite, carbon nanotubes, alumina nanocomposite, porous nanocomposite, mechanical properties, electrical properties

## Abstract

A novel tweakable nanocomposite was prepared by spark plasma sintering followed by systematic oxidation of carbon nanotube (CNT) molecules to produce alumina/carbon nanotube nanocomposites with surface porosities. The mechanical properties (flexural strength and fracture toughness), surface area, and electrical conductivities were characterized and compared. The nanocomposites were extensively analyzed by field emission scanning electron microscopy (FE-SEM) for 2D qualitative surface morphological analysis. Adding CNTs in ceramic matrices and then systematically oxidizing them, without substantial reduction in densification, induces significant capability to achieve desirable/application oriented balance between mechanical, electrical, and catalytic properties of these ceramic nanocomposites. This novel strategy, upon further development, opens new level of opportunities for real-world/industrial applications of these relatively novel engineering materials.

## 1. Introduction

**Figure 1:**
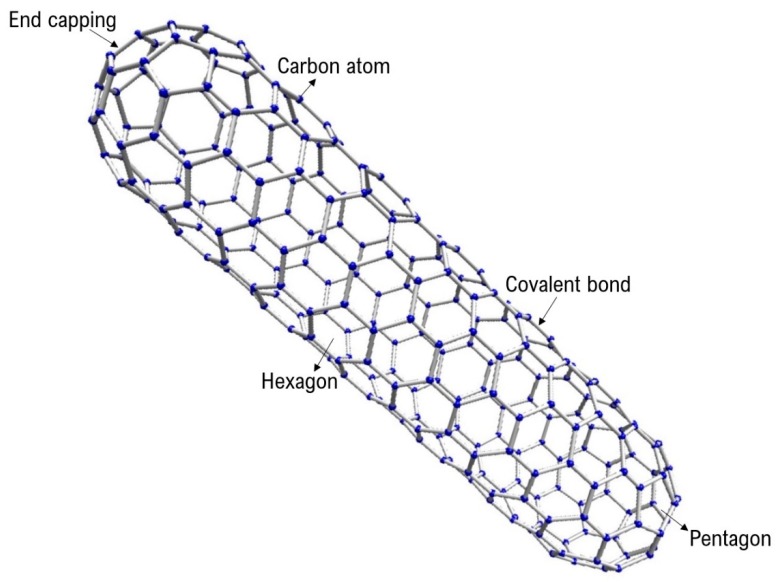
Carbon nanotube (CNT) molecule.

Carbon nanotubes (CNTs) are hollow cylindrical molecules (Figure 1) that consist of two-dimensional hexagonal lattice of carbon atoms, bent and joined in one direction [1]. Owing to their superlative combination of high surface area, large aspect ratio and excellent thermal, electrical and mechanical properties, CNTs have received significant interest among ceramic researchers [2,3,4,5,6,7]. In particular, for alumina–CNT nanocomposites, improved electrical [2], mechanical [3,4,5], and thermal [6] properties and utilization of CNTs for grain refinement and sintering aid [7] have been reported. 

CNTs, however, have not been analyzed extensively for their potential in ceramic nanocomposite membranes and/or porous structures to an extent where real-world/industrial applications could span out. In general, CNTs were impregnated into membranes or grown on structural materials for processing possible new types of filters and membrane technologies [8,9,10,11,12]. In terms of the ceramic filters, CNTs grown on micromachined Si/SiO2 [13] and glass fibers [14] have already demonstrated good filtration efficiencies. Addition and then subsequent modification of CNTs on alumina pores/porous structures has been the most common and widely explored method for producing ceramics nanocomposite membranes [15]. To analyze the filtration properties of these nanocomposites, Parham et al. grew CNTs on porous ceramic matrix consisting of mainly alumina and silica and demonstrated a high efficiency of yeast filtration (98%), around 100% for heavy metal ion removal from water and excellent particulate filtration from air [16]. The same group also grew CNTs in another study on porous alumina and reported higher compression strength for the porous alumina nanocomposites [17].

All of these previously published research efforts on alumina–CNT nanocomposites yielded mechanically weak filter structures when compared with bulk/monolithic alumina materials. Diffusion properties and catalytic activity of ceramic membranes/filters are normally tailored via changes in pore geometry, volume, and surface chemistry. This enables change in catalytic performance or the sorption capacity for a specific gaseous molecule [15]. However, in this report, we demonstrate a simple approach to produce strong, highly versatile and efficient alumina–CNT nanocomposite structures by manipulating surface porosity via systematic oxidation of CNTs at the grain boundaries. Such approach has not been explored nor demonstrated prior to this report, to the best of the authors’ knowledge. Here, we present a simple customization of CNT content after sintering, yielding desirable and application-oriented balance between mechanical, electrical, and catalytic properties. This is the first report which demonstrates such customization feature for superlative CNT filled ceramic nanocomposites.

## 2. Materials and Methods 

High purity (99.9%) multiwall CNTs (Ossila M2009D1 with outer diameters of 15 nm and lengths ranging 10–30 µm) were dispersed in dimethylformamide, DMF [18] using high power tip ultrasonication for 45 minutes and then hand-mixed with alumina nanopowder (Sigma–Aldrich, London, UK: gamma phase; particle size <50 nm; surface area 35–43 m^2^ g^−1^; melting point 2040 °C; and density 3.97 g cm^−3^) for 10 minutes. The liquid mixture was rotation ball milled for 10 h. It was then dried at 70 °C for 12 h using a rotary drier containing milling media (4 mm alumina balls), followed by vacuum oven drying at 100 °C for 50 h. To avoid re-agglomeration of CNTs during lengthy drying, the alumina balls (milling media) were added during rotary drying. The dried nanocomposite powder was ground and sieved at 150 mesh and then placed again in the vacuum oven at 100 °C for another 50 h to thoroughly remove the solvent from the mixed powder.

Monolithic alumina and alumina–5 wt% CNT nanocomposite pellets (diameter 20 mm and thickness 5 mm) were prepared by spark plasma sintering (SPS) using LABOX 350 (Sinter Land Inc, Nagaoka, Japan) furnace in vacuum to prevent the loss of CNTs during sintering. A pressure of 100 MPa was applied concurrently with the heating (rate 60 °C min^−1^) and released at the end of the sintering period, which was 10 min. Sintering temperature for all nanocomposites was 1250 °C. A pulsed DC current with 5 µsec ON and 5 µsec OFF was used without any pause. 

All of the sintered samples were ground down to 4000 grit using a SiC paper. Relative blackness (color intensity) after oxidation was quantified using Alicona Infinite focus microscope via Adobe Photoshop CC2015 software. The densities of the ground samples were measured using the Archimedes’ water buoyancy method and verified by a manual Heliulm multipycnometer (Quantachrome UK). All samples were then thoroughly dried in an oven at 80 °C for 24 h for removal of any moisture. All nanocomposite samples were then oxidized (heating rate 10 °C min^−1^) in an SNOL 3/1100 LHM01 laboratory furnace at 600 °C for various durations. The samples were cooled in an open furnace after the holding duration was completed and then diamond macro polished using 10 micron paste. The monolithic alumina and oxidized nanocomposite samples were then examined in a field emission scanning electron microscope (FE-SEM). The oxidized and polished surfaces were gold coated and observed in an ultra-high resolution analytical FE-SEM (Hitachi, SU-70) using a 20 keV electron beam. A separate batch of monolithic and nanocomposite samples were diamond polished and thermally etched at 1550 °C for 10 min for measuring grain sizes. Using FE-SEM, grain sizes were characterized after sintering and oxidation for monolithic alumina and nanocomposites respectively by the linear intercept method [19] using Equation (1).

D = 1.56 (L/MN)(1)

where D is the average grain size, L is the total length of test line used for calculation, N is the number of intercepts, and M is the magnification of the photomicrograph. About 660 intercepts were taken in consideration for each measurement. 

For all mechanical and electrical characterizations, at least seven samples of each composition were examined for greater confidence. Fracture toughness characterizations were carried out for alumina and nanocomposite samples according to standard ASTM C1421 (standard test method for determination of fracture toughness of advanced ceramics at ambient temperature). Single Edge V-Notch Beam (SEVNB) method was employed using parallelepiped samples (3 mm × 4 mm × 30 mm) and a loading span distance of around 17 mm. All samples were machined and notches were produced using a diamond saw (Accutom-50). For all samples, the notch was in the range of 0.7–1.1 mm in depth and around 195 µm in width. The root radius of the notch for each sample was about 9–10 µm with a V-notch angle of around 19°. The flexural strength was examined by three-point bending test. The testing was performed using a DS-II multifunctional desktop tester with a cross-head speed of 0.04 mm min^−1^ for enhanced accuracy. The flexural strength *FS* was calculated using Equation (2) [20]:

FS = 3PL/2bh^2^ (2)

where P is the load at the fracture point, L is the span length, b is the sample breadth, and h is the sample thickness.

For evaluating electrical conductivity, a bar (dimensions 17 mm × 3 mm × 5 mm) was cut from each sintered oxidized nanocomposite pellet using precision and deformation-free cutting machine (Accutom-50). Around 500 microns of material was removed from all surfaces of sample by fine grinding. Four-point method was employed by using a resistivity/Hall measurement system (Quantum Design, PPMS, Model 6000) for measuring the electrical conductivities of the nanocomposites. For pure alumina samples, high resistance meter (HP 4329A) was used to measure the conductivity. The connecting wires in the experimental setup were permanently bonded by using silver paste in order to avoid any contact resistance for this analysis. The specific surface area (SSA) was measured via Brunauer–Emmett–Teller (BET) theory using the Gemini 2360 analyzer, which is a fully automatic multi-point surface area analyzer utilizing flowing-gas technique. The technique used physical adsorption of nitrogen gas molecules on the solid surface of ceramic nanocomposite. All SSA measurements were carried out in accordance to ISO 9277:2010.

## 3. Results and Discussion

Monolithic alumina and alumina nanocomposite samples used in this study are presented in Table 1. The weight percentage of CNTs were 5% for all the nanocomposite samples. Relative blackness/color intensity is a semi-quantitative way of measuring the amount of left over CNTs after oxidation as well. It should be noted here that there is always some deposition of carbon from the carbon paper in contact with the powder during sintering, hence a relative scale was used for estimating the color intensities to give some qualitative understanding about the amount of carbonaceous content in relatively white/greyish pure alumina sintered pellet. Significant refinement in grain size was observed because of the addition of CNTs owing to a phenomenon reported previously as well [7]. CNTs have also been reported to support densification of alumina as observed in this work (Table 1) and previously published [7,21]. 

**Figure 2:**
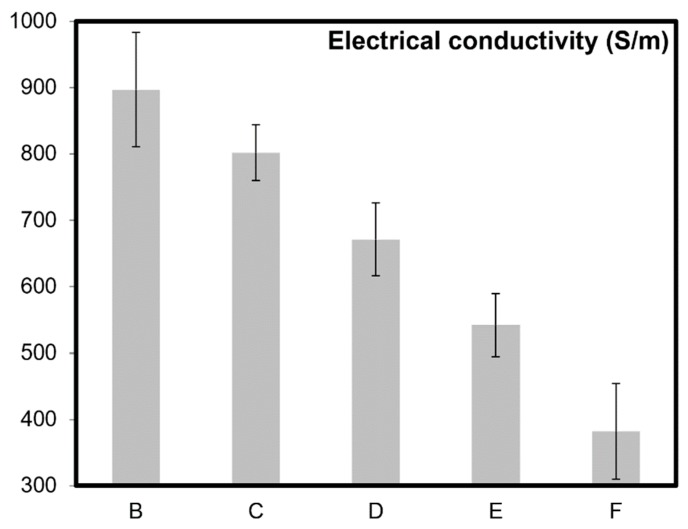
Electrical conductivity for alumina–5 wt% CNT nanocomposites measured by four-point method. Sample A (monolithic) is not included here as it is electrically non-conductive with a measured value of electrical conductivity in the order of 10^−13^ S/m.

**Figure 3:**
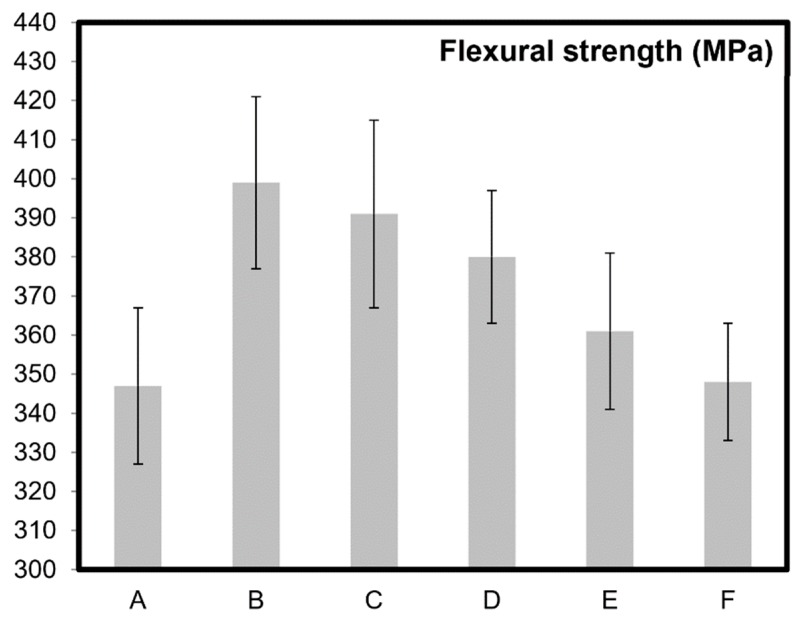
Flexural toughness of alumina (sample A) and alumina–5 wt% CNT nanocomposites (samples B–F). Respective oxidation times are: sample B (0 min), sample C (5 min), sample D (10 min), sample E (15 min), and sample F (20 min).

**Figure 4:**
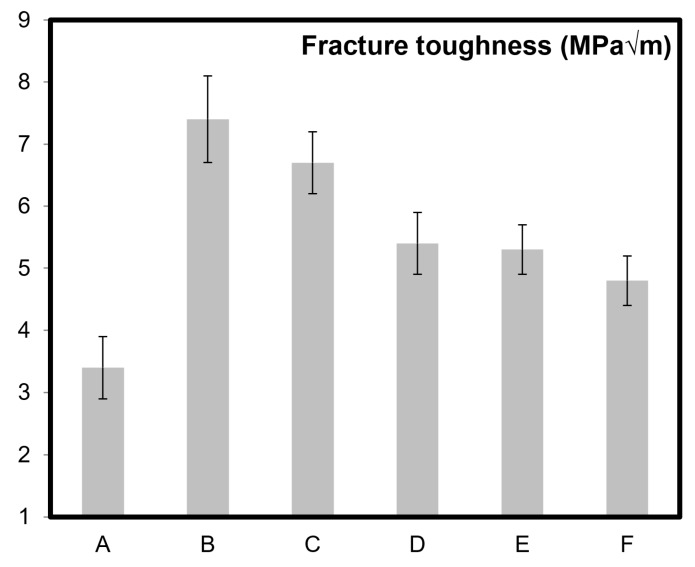
Fracture toughness of alumina (sample A) and alumina–5 wt% CNT nanocomposites (samples B–F). Respective oxidation times are: sample B (0 min), sample C (5 min), sample D (10 min), sample E (15 min), and sample F (20 min).

Figure 2, Figure 3 and Figure 4 present electrical and mechanical properties of the samples A–F described in Table 1. A clear variation between the theoretical density, electrical conductivity, and mechanical properties can be noted in Table 1 and from the analysis of Figure 2, Figure 3 and Figure 4. The emphasis prior to this study has always been on increasing the mechanical and electrical properties of these nanocomposites by varying the content of CNT molecules during dispersion/homogenization stage and prior to sintering. The oxidation (in air) of CNTs after sintering resulted in burn-out or removal of CNTs from the nanocomposite samples as reported elsewhere as well [21]. 

Surface area is a major aspect in catalysis and related technologies [16,17]. Apart from the chemistry, the surface area of a catalyst is responsible for affecting the rate of reaction. It is well understood that chemical reactions involving a catalyst occur on the surface of the catalyst and the catalyst works by lowering the overall activation energy of the process or reaction. CNT molecules-supported catalysts have also been widely explored in various research efforts [16,17]. Comparing sample B and F, it can be observed that a surface area of more than seven times was available in sample F (Table 1) without significant drop in mechanical properties (Figure 3 and Figure 4) as such. CNTs reside at the grain boundaries of alumina in the nanocomposites which has been confirmed previously [2]. With the oxidation of CNT molecules or elimination from the gain boundaries in samples B to F, customizable surface area and properties can be achieved for specific applications as noted in Table 1. 

The addition of CNT molecules for improving electrical conductivity of insulating materials like polymers [22,23] and ceramics [2,24] is widely appreciated. The axial electrical conductivity of CNTs was reported to be extremely high, reaching 2 × 10^7^ S/m [25], comparable to that of silver, copper, gold, and aluminum (10^7^ S/m) [26]. The electrical conductivity of alumina–CNT nanocomposite increased with decreasing grain boundary area because of the increased number of conductive pathways formed by the CNT molecules [2]. This is also due to the CNTs’ fibrous nature/high aspect ratio and very high electron mobility within CNTs [2]. It can be observed from Figure 2, that the electrical conductivity decreases with the increase in oxidation temperature because of the removal of the electrically conductive elements from the nanocomposites. For instance, a reduction of 57% was observed in the electrical conductivity of nanocomposites with just 20 minute of oxidation time after sintering and achieving full densification. It should be noted here that the burn out of CNT molecules is also responsible for slightly lowering the densification as well, as reported in Table 1. 

From the application perspectives, ceramics with such tailorable or customizable electrical conductivities have many industrial applications, such as, ceramic heaters (and other high-temperature/thermoelectric applications), electric discharge machining (EDM), static charge dissipation, batteries, lightning protection, electromagnetic interference (EMI) shielding in electronic, mechanical, structural, chemical, and vacuum applications [2,27]. In particular, alumina with added electrically conductive fillers has been used to fabricate substrates for handling semiconductor wafers that require static protection [27]. The present research demonstrated the strategy of post-sintering oxidation and initiating removal of intertangled networks of CNTs and yielding lower electrical conductivities for the bulk nanocomposite, upon further development, could offer significant customization to these applications.

Like electrical properties (Figure 2), similar trend can also be noted for the mechanical properties (Figure 3 and Figure 4). All the previous research and attempts to modify such properties in CNT based ceramics primarily focused on the homogenization strategies and volume content of CNT molecules. This report discusses a simple but effective post-sintering strategy where a desirable balance between mechanical, electrical, and catalytic properties of the materials can be achieved by careful removal of CNTs from the grain boundaries of ceramic matrices. For example, to achieve a certain portfolio of mechanical properties, the possibility of lowering flexural strength and fracture toughness up to 13% and 35% respectively in these nanocomposites have been demonstrated in Figure 3 and Figure 4. For monolithic ceramics, there has always been a strong dependency of mechanical properties like flexural strength and fracture toughness on the relative density of ceramics [28,29]. However, because of the presence of CNTs at the grain boundaries of alumina, such dependency is significantly minimized as observed in Figure 3 and Figure 4. It should be noted here that the grain refinement [21], crack pinning and bridging mechanisms [2,30,31] in these nanocomposites are attributing to these key observations. In general, and as widely appreciated [2,3,4,5,6], the superior electrical and mechanical properties reported in Figure 2, Figure 3 and Figure 4 are due to the high aspect ratios and intrinsic chicken wire (strong sp^2^ covalent bonds) structure (Figure 1) of CNTs [1].

**Figure 5:**
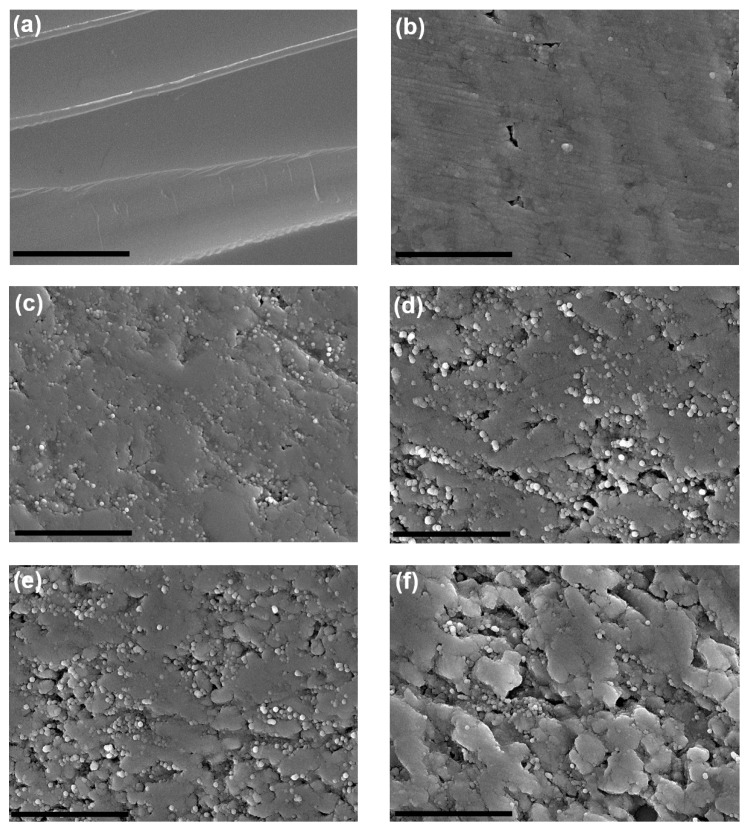
Representative FE-SEM images for macro polished alumina and alumina–5 wt% CNT nanocomposites: (**a**) monolithic alumina; alumina nanocomposites with oxidation durations of: (**b**) 0 min; (**c**) 5 min; (**d**) 10 min; (**e**) 15 min; and (**f**) 20 min. Scale bar represents 100 nm.

Field emission scanning electron micrographs of polished and oxidized materials are presented in Figure 5. CNT molecules intrinsically possess graphitic lubricating surface properties [7,32,33]. A clear difference in surface morphology can be observed between monolithic alumina and nanocomposite sample (Figure 5a–c). From the comparative analysis of Figure 5b–f, it can be observed that longer the oxidation durations, higher the grain pluck outs after macro polishing and vice versa. CNTs create an intertangled network of strong nanowires which hold all the alumina grains in place. Upon removal of CNT molecules from the grain boundaries and because of their lubricating surface properties, it is easier to create more uneven surfaces as can be seen from Figure 5f. Also, from these representative FE-SEM images (Figure 5c–f), a good level of homogenized distribution of CNTs can be evidenced. Such good and uniform levels of homogenization is essential for utilization of these structures in catalytic applications [34].

The existence of CNTs and other forms of fullerenes after sintering were confirmed in our previous studies [35,36]. CNT molecules were found to be well preserved in alumina after sintering up to 1900 °C and with a pressing pressure of 100 MPa [36]. The results via high resolution electron microscopy, X-ray diffraction, and Raman spectroscopy [36]. In the same study, multiwall carbon nanotubes maintained their high aspect ratio and fibrous nature even after being sintered in boron carbide at 2000 °C for 20 min. It should also be noted here that the existence of good quality CNT molecules in nanocomposites (after sintering) can also be indirectly confirmed by enhancement of electrical and mechanical properties, where results/values reported in Table 1 and Figure 2, Figure 3 and Figure 4 are comparable to those already reported in literature [2,3,4]. 

To recapitulate briefly, adding CNT molecules in ceramic matrices and then systematically oxidizing them induces significant capability to customize mechanical, electrical, and catalytic properties of these materials (Figure 2, Figure 3 and Figure 4). Oxidation of CNTs, an irreversible process, offers a post sintering manipulation capability to ceramic nanocomposites. The present study enables tailoring capability by achieving a good balance between the specific surface area of CNT molecule supported materials and their bulk structural mechanical and electrical properties. Further development of this strategy for these superlative carbon based materials would lead toward their practical applications.

## 4. Conclusions

The post-sintering manipulative nature of CNT molecules-based ceramic nanocomposites is demonstrated in this work. Such customization of key electrical and mechanical properties has always been reported by changing the carbonaceous content in these nanocomposites in all of the previous reports. This work demonstrates a simple but very effective strategy to tailor the surface area, mechanical properties (flexural strength and toughness), and electrical conductivity of ceramics for desired combination of properties using appropriate oxidation durations. A strong dependency of CNT content on surface area, mechanical properties (flexural strength and toughness), and electrical conductivity was observed which could be manipulated after sintering as demonstrated here. The ability to change these properties after sintering and without compromising on the structural integrity opens new opportunities for real-world/industrial applications for this family of relatively novel engineering materials.

## Figures and Tables

**Table 1:** Alumina and alumina–CNT nanocomposites used in this work.

Sample Name	wt% of CNTs	Grain Size, Before Oxidation	Oxidation Time (mins) After Sintering	Relative Color Intensity After Oxidation (%)	Theoretical Density (%), After Oxidation	Specific Surface Area (m^2^/g)
A	0	5.3 ± 1.7 µm	NA	0	NA	1.9 ± 0.2
B	5	78 ± 36 nm	0	100	99.9	4.9 ± 0.5
C	5	84 ± 32 nm	5	92	99.5	15.3 ± 0.2
D	5	90 ± 43 nm	10	80	99.0	22.9 ± 0.3
E	5	85 ± 65 nm	15	72	98.7	26.3 ± 0.6
F	5	89 ± 46 nm	20	61	98.5	37.9 ± 0.7
